# Infantile Mortality in Workhouses

**Published:** 1909-05-08

**Authors:** Beatrice Webb

**Affiliations:** 41 Grosvenor Road, Westminster


### EDITOR'S LETTER-BOX.

### INFANTILE MORTALITY IN WORKHOUSES.

*To the Editor of* THE HOSPITAL.

SIR,—The Local Government Board has taken an unusual step. It has published as a Parliamentary Paper a defence of the workhouse nurseries, seeking to show that their condition is satisfactory, and that there is no greater mortality among the babies born than may properly be expected. The careless reader, like the complacent guardian of the poor, may easily suppose that the things which the Commissioners have themselves personally seen in the workhouse nurseries, like the damaging evidence of the official reports themselves, are all a delusion, and that the workhouse nursery is the best possible.

But let us look a little closer at this "whitewashing" report. Notwithstanding the appeal that we made for official statistics of the ten or twelve thousand babies annually born in the workhouses, the only figures now vouchsafed relate to London alone. So far as they go these are actually confirmatory of the statistics of the Minority Report. We stated that out of 8,483 babies born in 1907 in 450 workhouses, 1,050 died on the premises within that year, being 12.4 per cent. of the whole (all readmissions being, by the way, excluded); and that the deaths during the first week were from 40 to 45 per 1,000 births. The figures which Mr. Burns has been persuaded to publish show that out of 2,653 children born in Metropolitan workhouses during 1907, 312 died on the premises within twelve months of birth, including readmissions, being 11.7 per cent. of the whole; and that the mortality during the first week was 42.2 per 1,000 births—admittedly "nearly double the corresponding rate obtaining among the general population." If this is the case in the Metropolitan infirmaries, what is it likely to be in the rest of the Kingdom, where (with some conspicuous exceptions) the general mixed workhouses are older, less well-equipped, and less well-staffed than the London infirmaries? Moreover, what is the use of publishing testimonies to the excellence of the lying-in wards, when what is in question is the state of the infant nurseries? I challenge Mr. Burns to publish exact statistics of all the births in the Poor-law institutions of England and Wales for the past five years, together with the corresponding infantile deaths on the premises at each period of life in such a way as to be comparable with the Registrar-General's figures for the general population.

But the Local Government Board is apparently quite satisfied that the babies in the workhouse nurseries—with ample food and nursing, and constant attendance—should die at twice the rate of the babies of the general population, exposed to all the risks and hazards of the average household. The excess is apparently to be fully accounted for by "pre-natal and maternal conditions." This argument would have sounded equally valid if four-fifths of the babies had died! Unfortunately, however, it does not account for the startling differences in infantile mortality between adjoining unions in the same town (not, by the way, as is disingenuously suggested, between urban and rural populations). It may be difficult to keep down the infantile mortality of a London workhouse lying-in ward (average 42.2 per 1,000 for the first week) to the rate of the Rotunda Hospital at Dublin, where it is less than a quarter of this figure. But how is it that in one workhouse nursery ten times as many babies die per 1,000 births as in another? Can there be all this difference between the "pre-natal and maternal conditions" of the women coming in to be confined; and are we to take it for granted that these differences accurately correspond with the varying infantile death-rates of the several workhouses? I cannot help thinking that the L.G.B. statisticians know better than that.

It is, indeed, an interesting fact that some of the workhouse nurseries (including some dealing with the poorest slums of London and other large towns) have, with all the terrible "pre-natal and maternal conditions," actually a lower infantile mortality than prevails in the general population. I feel that, by giving in the Minority Report only the average for the whole, we failed to do proper justice to these excellent unions, which have overcome so many difficulties; and at the same time we understated our case. Why is it that such unions (to take only some of the good cases) as Stepney, Camberwell, Marylebone, St. George's in the East, and St. George's, Hanover Square, in the Metropolis, or Ashton-under-Lyne, Stockport, and Prescot, in the manufacturing districts, can all show infantile death-rates comparable with those of the best maternity hospitals, and considerably lower than those of the general population; whilst other unions of the Metropolis not poorer than Stepney or St. George's in the East, and unions in provincial towns having populations closely resembling those of the industrial towns mentioned, have infantile death-rates six, eight, and ten times as great? If the adverse influence of the "pre-natal and maternal conditions" has been overcome in some unions, how is it that other workhouse nurseries actually in the same town, or dealing with quite similar populations, kill off their babies at ten times the rate; and have been allowed to do so, year after year, without criticism or rebuke? Unhappily the "whitewashing" Memorandum now published will encourage these bad unions in their self-complacency.

To my untutored mind—I ask any mother whether I am not right in this?—the following facts (none of which are refuted or denied) have at least something to do with it.

1. In some workhouse nurseries the babies never get into the open air from the time they are born until they die, or leave at three or four or five. This fact seems incredible, but the L.G.B. does not deny it. In fact, it sanctions placing the nursery on a fourth story, without even balconies.

2. Whilst the nursing staff is slowly improving, in most nurseries of general mixed workhouses the babies are still handled, in the main, by pauper women (this not yet being forbidden by the L.G.B.), many of them feeble-minded, and occasionally even imbecile.

3. In some workhouse nurseries not even one trained nurse is in charge (this not yet being required by regulation).

4. There is no "quarantine" of newcomers; a perpetual stream of infants from 0 to 4 pours in from the worst home conditions without a probation ward, straight into the nursery. No wonder that measles and whooping cough are "troublesome"!—I am, etc.,

BEATRICE WEBB (Mrs. Sidney Webb).

41 Grosvenor Road, Westminster.

